# Supplementary material for: Lateral recurrence dynamically refines visual representations for task performance
Source: bioRxiv. 2026 Oct 9:2026.10.02.756219. Preprint. [Version 1] doi: 10.64898/2026.10.02.756219 (PMC13692027; doi:10.64898/2026.10.02.756219)
Supplement: Supplementary file 1 [file NIHPP2026.10.02.756219V1-supplement-1.pdf]

bioRxiv preprint doi: https://doi.org/10.64898/2026.10.02.756219; this version posted October 9, 2026. The copyright holder for this preprint (which was not certified by peer review) is the author/funder, who has granted bioRxiv a license to display the preprint in perpetuity. It is made available under a CC-BY 4.0 International license.

Supplementary Information

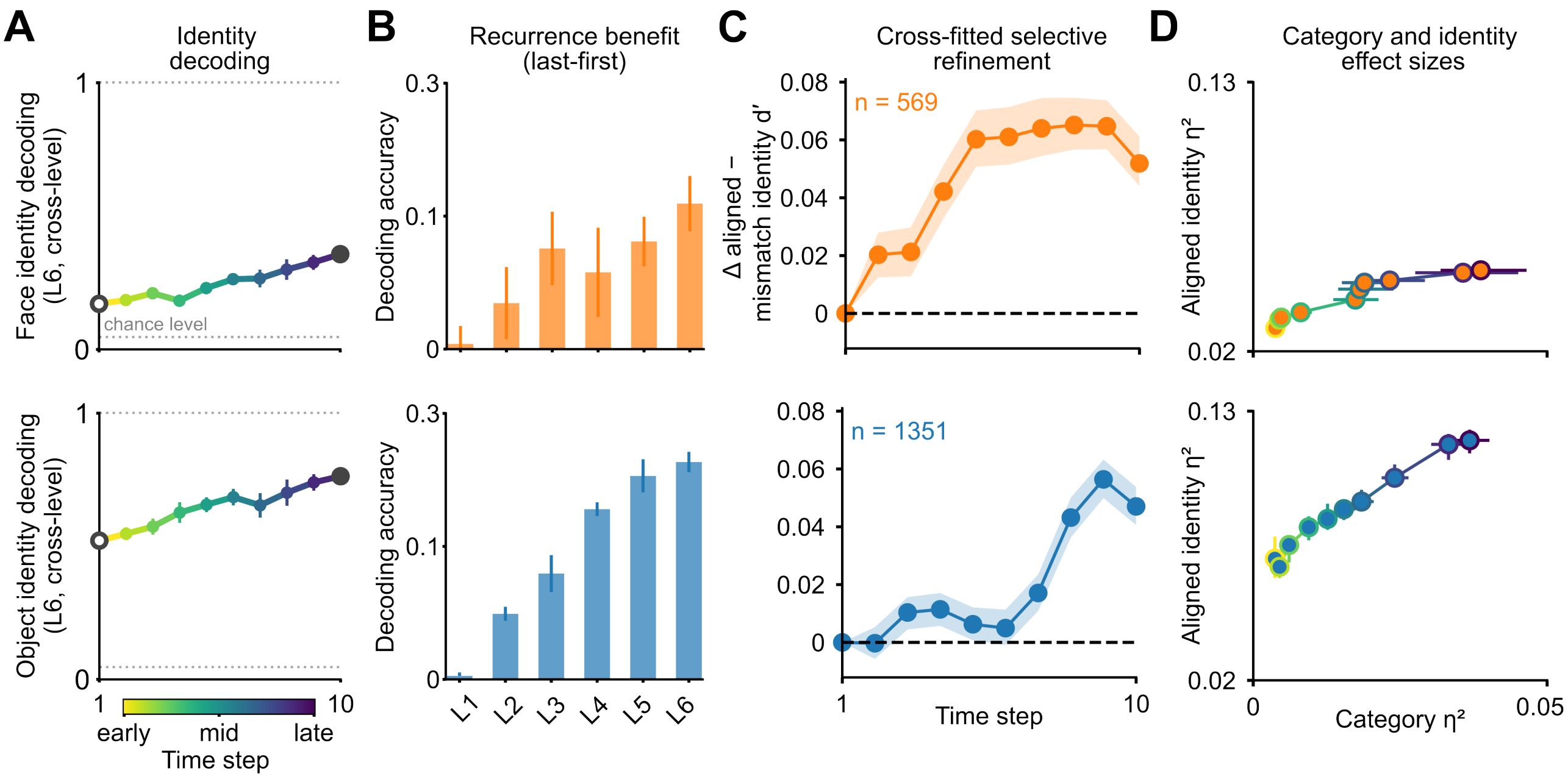

Figure S1. Population- and unit-level analyses of recurrent processing in the jointly-trained model. Analyses for the model trained on both faces (VGGFace2) and objects (ImageNet). Top row shows results for face-selective units; bottom row shows results for object-selective units. (**A**) Population decoding across recurrent time. Curves show decoding performance for the corresponding preferred domain across recurrent steps; points are colored by time step (early to late, color bar below). The dotted horizontal line indicates chance level. (**B**) Population recurrent gain across layers, computed as the late-minus-early change in decoding accuracy for each layer. Bars show mean gain and error bars indicate SEM across leave-one-level-out viewpoint splits. (**C**) Cross-fitted single-unit selective-refinement time course. Units were first classified as face- or object-selective using category d' computed on one split of the data, and aligned and mismatched identity d' were then measured on the held-out split. Selective refinement was defined as aligned minus mismatched identity d', where aligned identity d' corresponds to face identity d' for face-selective units and object identity d' for object-selective units. Values are plotted as the change relative to the first recurrent time point, so positive values indicate an increase over time in identity sensitivity aligned with the unit's category preference. Lines show the mean across units, shaded bands show bootstrap 95% confidence intervals, and the number of stable units included in each analysis is indicated in each panel. The smaller number of face-selective units reflects the higher class count and harder optimization landscape of fine-grained face identification relative to object categorization. (**D**) Single-unit variance decomposition for the same stable units. Trajectories plot category η^2 against aligned identity η^2 across recurrent time, with points colored by time step. Error bars show bootstrap 95% confidence intervals across units. Together, these analyses show that recurrent processing strengthens category-aligned identity information in both face-selective and object-selective subpopulations within a single jointly-trained network. The same coupling between category preference and identity refinement therefore emerges within one model and one training regime, generalizing the dissociation observed in the single-task models.

bioRxiv preprint doi: https://doi.org/10.64898/2026.10.02.756219; this version posted October 9, 2026. The copyright holder for this preprint (which was not certified by peer review) is the author/funder, who has granted bioRxiv a license to display the preprint in perpetuity. It is made available under a CC-BY 4.0 International license.

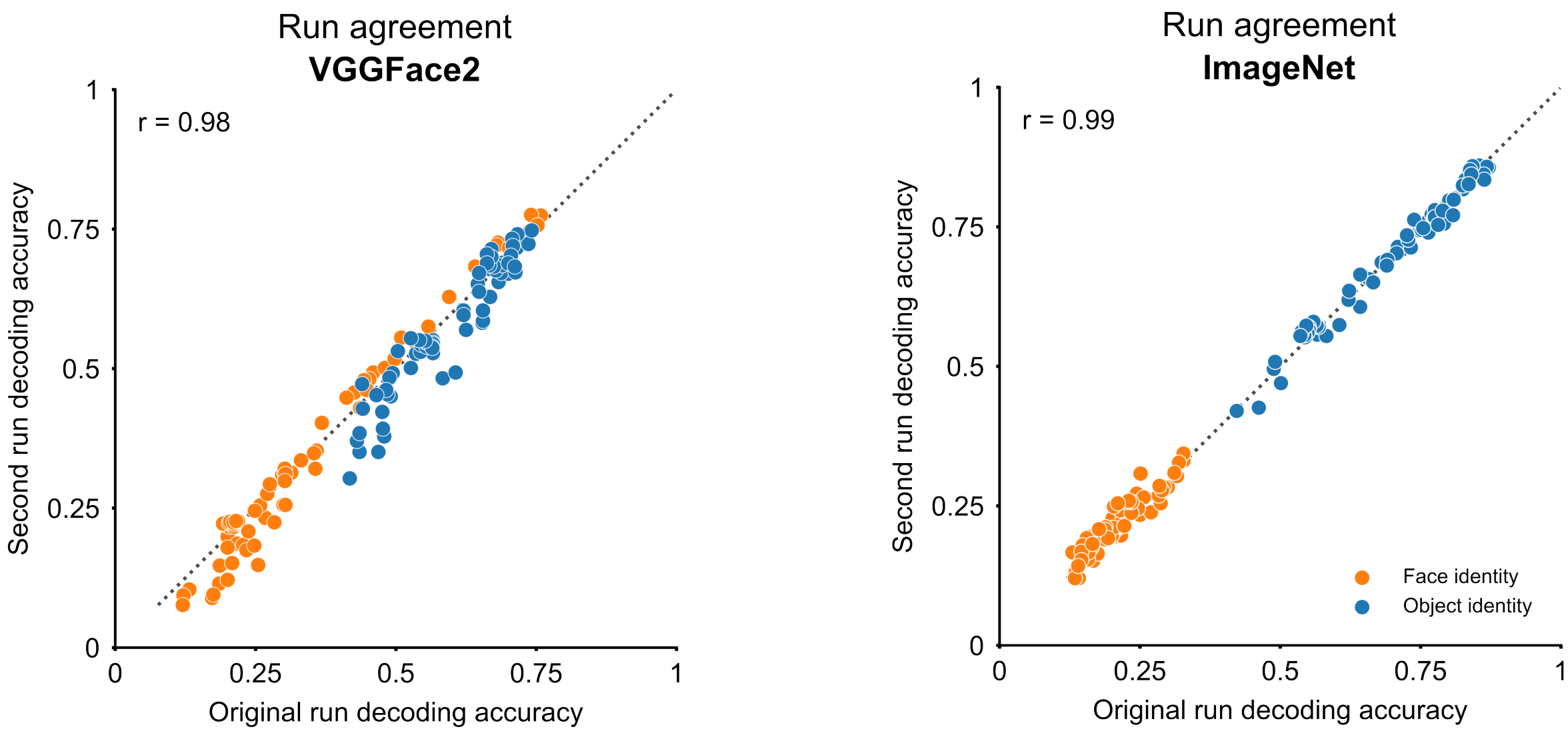

Figure S2. Reproducibility of decoding results across independent training runs. Decoding accuracy from the original training run (x-axis) is plotted against decoding accuracy from a second independent training run with a different random initialization (y-axis), separately for the VGGFace2-trained model (left; r=0.98) and the ImageNet-trained model (right; r=0.99). Each point corresponds to one (layer × recurrent timestep) combination (n=75). Points fall close to the unity line (dotted), indicating that the patterns reported in the main text are stable across independent training runs. Orange: face-identity decoding; blue: object-identity decoding.

bioRxiv preprint doi: https://doi.org/10.64898/2026.10.02.756219; this version posted October 9, 2026. The copyright holder for this preprint (which was not certified by peer review) is the author/funder, who has granted bioRxiv a license to display the preprint in perpetuity. It is made available under a CC-BY 4.0 International license.

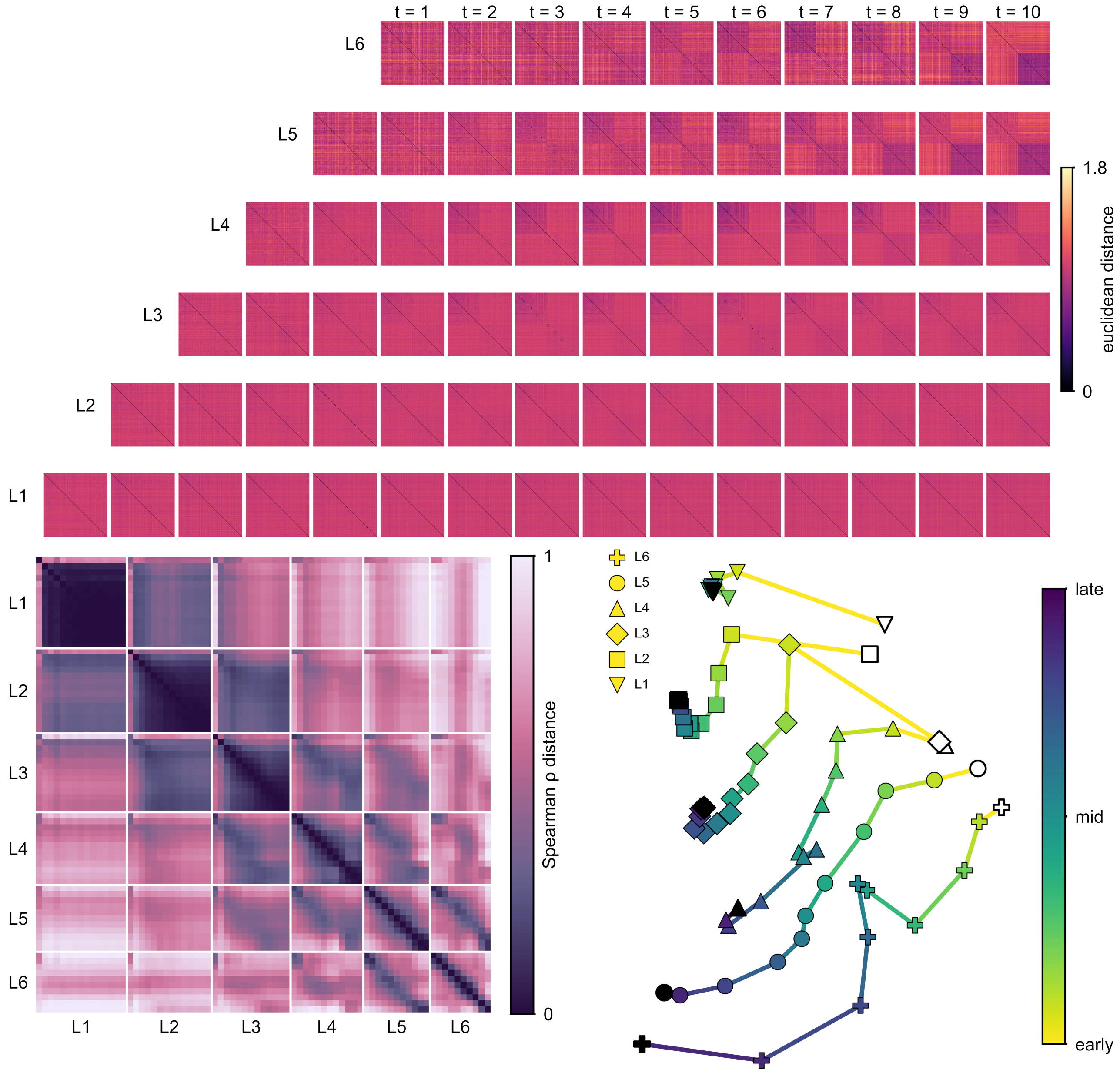

Figure S3. Population geometry results replicate under Euclidean distance. Identical analyses to Figure 3, but with first-order representational dissimilarity computed using normalized Euclidean distance rather than correlation distance. Pairwise Euclidean distances were computed between population response vectors, normalized by the square root of the number of units in the layer to control for differences in dimensionality, and then divided by the mean off-diagonal distance within each RDM so that color values represent dissimilarity relative to each RDM's average. Color values therefore indicate within-RDM relative structure rather than absolute distances, which would otherwise be confounded by timestep-to-timestep fluctuations in overall response magnitude inherent to recurrent dynamics. (**A**) RDMs across layers and recurrent timesteps, organized as in Figure 3A. (**B**) Second-order Spearman ρ distance matrix between RDMs from each layer–timestep combination. (**C**) MDS embedding of the second-order distance matrix. The temporal evolution of representational geometry, the stability of early-layer representations, and the larger displacement of mid-to-late layers across recurrent time all replicate the patterns observed under correlation distance (Fig. 3), confirming that the main conclusions are robust to the choice of distance metric.

bioRxiv preprint doi: https://doi.org/10.64898/2026.10.02.756219; this version posted October 9, 2026. The copyright holder for this preprint (which was not certified by peer review) is the author/funder, who has granted bioRxiv a license to display the preprint in perpetuity. It is made available under a CC-BY 4.0 International license.

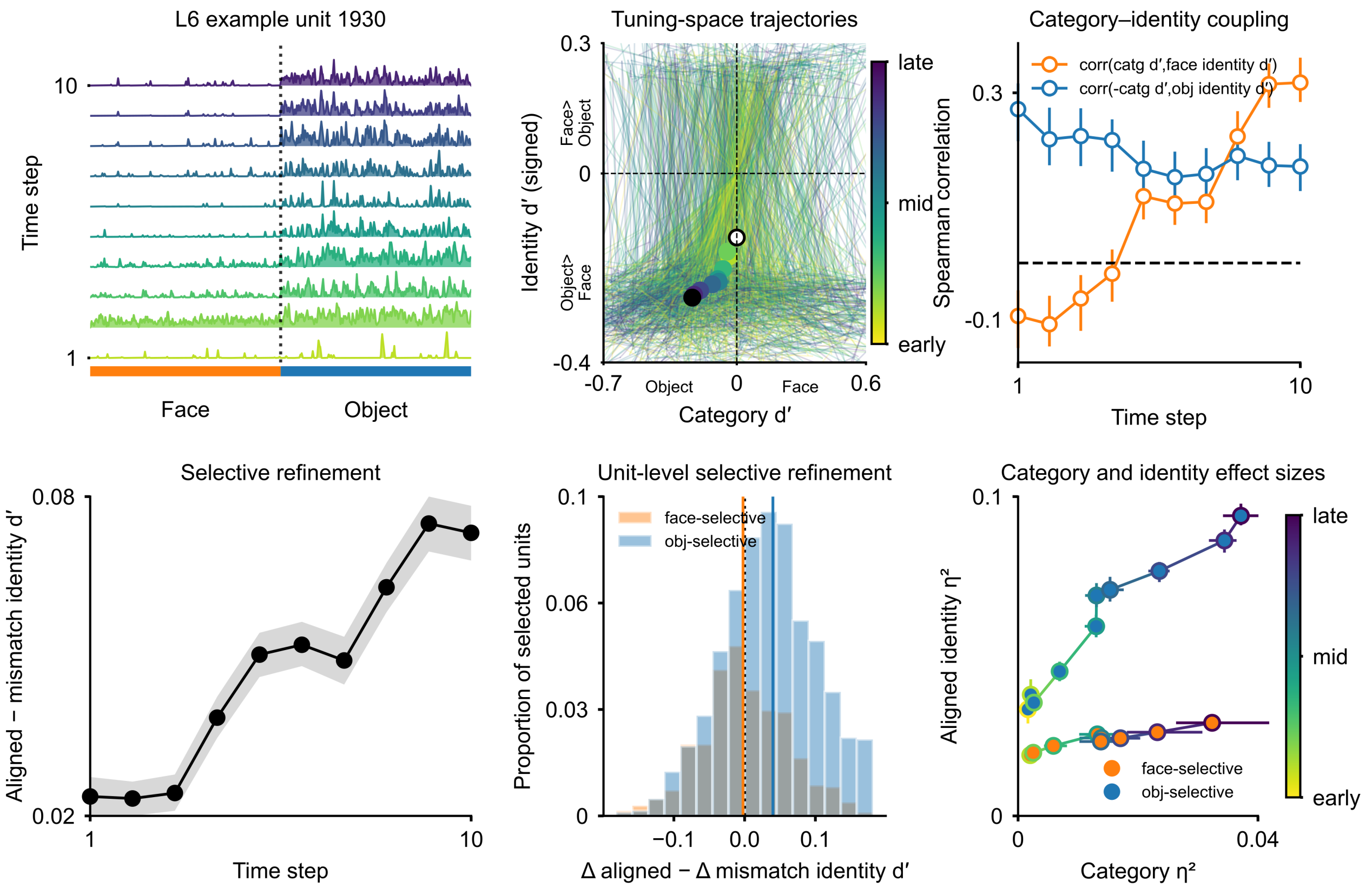

Figure S4. Recurrent computations strengthen both object-category selectivity and object-identity discrimination in the same units in the object-recognition-trained model. Panels follow the same layout as Fig. 6, computed here for the object-trained model. (**A**) Response profile of an example L6 unit (unit 1930) across recurrent timesteps. (**B**) Trajectories of individual L6 units in the tuning space defined by category d′ and signed identity d′. (**C**) Spearman correlations across L6 units between category d′ and face-identity d′ (orange), and between negative category d′ and object-identity d′ (blue), at each recurrent timestep. (**D**) Average selective-refinement index across units (aligned minus mismatched identity d′; cross-fitted, see Methods). (**E**) Distribution of unit-level changes in selective refinement from early to late recurrence, for face-selective (orange) and object-selective (blue) units. (**F**) Variance decomposition, plotting category η^2 against aligned identity η^2, colored by recurrent timestep. Across timesteps, object-selective units gain object-identity discrimination while preserving category tuning, mirroring the face-trained model with the domains reversed. Conventions and statistics as in Fig. 6.
